# Exploring Mechanical Features of 3D Head and Neck Cancer Models

**DOI:** 10.3390/jfb16030074

**Published:** 2025-02-20

**Authors:** Aleksandra Evangelista, Franca Scocozza, Michele Conti, Ferdinando Auricchio, Bice Conti, Rossella Dorati, Ida Genta, Marco Benazzo, Silvia Pisani

**Affiliations:** 1 Department of Otorhinolaryngology, Fondazione IRCCS Policlinico San Matteo, Via Golgi 19, 27100 Pavia, Italy; a.evangelista@smatteo.pv.it (A.E.); m.benazzo@smatteo.pv.it (M.B.); 2 Department of Civil Engineering and Architecture, University of Pavia, Via Ferrata 3, 27100 Pavia, Italy; michele.conti@unipv.it (M.C.); ferdinando.auricchio@unipv.it (F.A.); 3 3D and Computer Simulation Laboratory, IRCCS Policlinico San Donato, Piazza Edmondo Malan 2, San Donato Milanese, 20097 Milano, Italy; 4 Department of Drug Sciences, University of Pavia, Via Taramelli 12, 27100 Pavia, Italy; bice.conti@unipv.it (B.C.); rossella.dorati@unipv.it (R.D.); ida.genta@unipv.it (I.G.); silvia.pisani@unipv.it (S.P.)

**Keywords:** HNSCC, 3D models, mechanical properties, 3D bioprinting, cancer models

## Abstract

Head and neck squamous cell carcinoma (HNSCC) presents significant challenges in oncology due to its complex biology and poor prognosis. Traditional two-dimensional (2D) cell culture models cannot replicate the intricate tumor microenvironment, limiting their usefulness in studying disease mechanisms and testing therapies. In contrast, three-dimensional (3D) in vitro models provide more realistic platforms that better mimic the architecture, mechanical features, and cellular interactions of HNSCC. This review explores the mechanical properties of 3D in vitro models developed for HNSCC research. It highlights key 3D culture techniques, such as spheroids, organoids, and bioprinted tissues, emphasizing their ability to simulate critical tumor characteristics like hypoxia, drug resistance, and metastasis. Particular attention is given to stiffness, elasticity, and dynamic behavior, highlighting how these models emulate native tumor tissues. By enhancing the physiological relevance of in vitro studies, 3D models offer significant potential to revolutionize HNSCC research and facilitate the development of effective, personalized therapeutic strategies. This review bridges the gap between preclinical and clinical applications by summarizing the mechanical properties of 3D models and providing guidance for developing systems that replicate both biological and mechanical characteristics of tumor tissues, advancing innovation in cancer research and therapy.

## 1. Introduction

Head and neck squamous cell carcinoma (HNSCC) ranks as the sixth most prevalent cancer worldwide. Primarily located in the oral cavity, larynx, and pharynx, it arises from the mucosal epithelium; these areas are adjacent to numerous critical structures, complicating the possibility of extensive surgical removal. This tumor is often diagnosed at a late stage, exhibits rapid progression, and shows resistance to antitumor treatment strategies [1]. Developing reliable preclinical models is crucial for gaining a deeper understanding of the molecular mechanisms underlying HNSCC treatment resistance and progression. These models are also crucial for identifying more effective therapeutic strategies.

Drug screening in monolayer cell cultures (2D in vitro model) remains the primary approach for discovering new therapeutic agents. Immortalized cell lines obtained from HNSCC tumors serve as an important tool for assessing the efficacy of antitumor treatments, exploring mechanisms of resistance, and enhancing early diagnostic and screening methods. The advantages of cell lines as the simplest in vitro tumor model are evident: they are easy to use, allow for precise control of external factors, are economically accessible, and have a wide range of applications [2]. However, three-dimensional (3D) in vitro models, which more accurately mimic the architecture and cellular environment of tumor tissue, may be superior in predicting drug efficacy and tumor progression. Studies using 3D cell cultures have demonstrated significant variations in radiation and drug sensitivity, akin to those observed in in vivo tumors [3]. The main advantages of 3D models compared to the traditional 2D are their ability to better mimic the complexity and behavior of primary tumors while 2D models fall short in replicating the physiological environment of living tissues. In contrast, 3D models, particularly those using cell lines or primary cells, provide a more accurate representation of the tumor microenvironment by promoting cell–cell and cell–extracellular matrix (ECM) interactions. This leads to enhanced cell proliferation, differentiation, and morphology that closely resemble in vivo conditions. However, 3D models come with challenges such as increased costs, more time-consuming examination processes, and technical difficulties in handling [4]. While the cost and technical demands of 3D models are acknowledged, they offer significant advantages in terms of predictive accuracy for tumor progression and therapeutic response. These benefits, particularly in drug screening and the reduction of animal testing, provide a strong justification for their adoption despite the higher initial investment. A comparative cost–benefit analysis would help to further demonstrate how these models could ultimately lead to more effective and personalized treatments, potentially offsetting costs in the long term [5].

Thus, robust preclinical models are necessary to investigate the mechanisms of treatment resistance and progression in HNSCC, which will help to refine and improve therapeutic strategies. In recent years, the development of patient-specific 3D cell models represents a significant advancement in HNSCC research, offering a tailored approach to testing chemotherapeutic drugs with greater specificity, enhancing our ability to combat treatment resistance and advance personalized therapeutic strategies for patients. These advancements help rationalize the use of animals in research, aligning with the principles of “the 3Rs” (reduction, replacement, and refinement) in animal experimentation [1,6,7].

**Figure 1:**
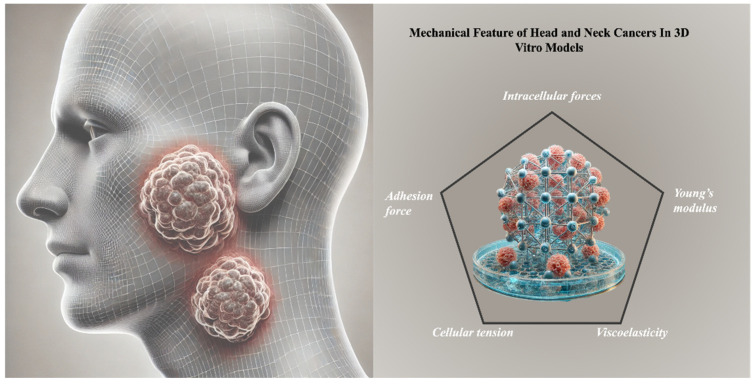
This review explores the mechanical features of head and neck cancers (HNCs) in 3D in vitro models aiming to enhance our understanding of tumor biomechanics with direct clinical applications. This approach facilitates the development of more effective therapies, enables testing of personalized treatments, and offers insights to improve surgical strategies.

This review aims to provide a comprehensive summary of the latest and most innovative 3D in vitro models developed for head and neck squamous cell carcinoma (HNSCC) research, with a particular focus on their mechanical properties (Figure 1). The development of 3D models still faces challenges, including standardizing mechanical properties across systems, reproducing the complexity of the native extracellular matrix, and scaling models for high-throughput screening. While research efforts have been initiated to address some of these challenges, such as refining mechanical tunability and enhancing matrix composition, others—such as the dynamic simulation of vascularization or immune interactions—remain largely unexplored. Highlighting these gaps more explicitly is essential to map the trajectory of future research. Addressing these limitations is essential to fully realize the potential of 3D in vitro models as transformative tools in cancer research [8].

Understanding and replicating in vitro the mechanical characteristics of the tumor microenvironment is crucial for enhancing the physiological relevance of these 3D models. These capabilities bridge the gap between conventional in vitro methods and in vivo studies, enhancing the predictive power of drug testing and accelerating the development of effective therapies for HNSCC. This review also explores how mechanical properties influence tumor growth, metastasis, and treatment response, providing direct clinical applications. By closely replicating the structural and biomechanical characteristics of head and neck cancers (HNCs), 3D models enable the development of more effective therapies, support personalized treatment testing, and offer insights to refine surgical strategies. Ultimately, this approach contributes to advancing therapeutic strategies and improving clinical outcomes for patients with HNCs [9].

## 2. Mechanical Properties of HNSCC Models

Cancer is a complex, multi-phase condition marked by abnormal regulation and uncontrolled cell growth, leading to the development of solid tumors. Mechanical abnormalities present within the tumor microenvironment (TME), including compressive forces and matrix stiffness, affect cancer cells at the molecular, cellular, and tissue level to fuel tumor growth and metastasis [10]. Studies show that examining the mechanical characteristics of cells and tissues in a developing tumor is essential for understanding how cancers maintain their molecular structure and organization. Various mechanical properties are altered in cancer cells, such as cell tension, hydrostatic pressure, adhesion strength, elasticity (Young’s modulus E), and viscosity (η) [11].

Among the many mechanical properties analyzed in mechanobiological studies, stiffness is one of the most frequently assessed metrics. It is typically measured using Young’s modulus, which represents the ratio of stress (σ), or the applied force per unit area, to strain (ε), which quantifies the relative change in length compared to the material’s original length [12]. However, only stiffness does not fully capture the true mechanical behavior of biological materials due to the highly heterogeneous nature of both cells and tissues, and these structures typically display a time-dependent response rather than linear-elastic deformation when subjected to an external force.

A related concept used in certain measurement systems is compliance (δ), which is the inverse of stiffness and represents the flexibility of a material.

Cancerous cells undergo significant changes in their cytoskeleton and plasma membrane, which profoundly affect the physical properties of the surrounding tissue. Research has consistently shown that these alterations lead to a general decrease in cellular tension, a phenomenon closely linked to malignant progression. One notable consequence of these structural changes is the reduced shear modulus observed in cancerous cells compared to healthy ones. By analyzing the mechanical properties of cancer cells and tissues (Table 1), scientists can gain valuable insights into how the modulation of specific biomolecules or pharmacological treatments might influence cancer cell aggressiveness—either by enhancing or suppressing it [11,13]. Furthermore, understanding these mechanical alterations plays a crucial role in the development of increasingly realistic and predictive 3D models.

The tumor microenvironment (TME) is a dynamic and complex system where both cellular and extracellular components interact to influence tumor growth, progression, and response to therapy. Among the factors shaping the TME, mechanical properties play a pivotal role, directly impacting processes such as cell migration, invasion, and drug resistance. These mechanical characteristics are not solely determined by the tumor cells but are also profoundly influenced by components of the TME, including integrins and stromal elements. Integrins, for instance, are significant therapeutic targets in squamous cell carcinoma (SCC). Among these, cilengitide, a specific integrin inhibitor, has been clinically evaluated in combination with 5-fluorouracil (5-FU), cisplatin, and cetuximab, an epidermal growth factor receptor (EGFR) inhibitor, in studies aimed at improving therapeutic efficacy [19].

However, the role of additional TME factors in HNSCC progression remains incompletely understood. The immune system, for example, plays a dual role in HNSCC, both promoting tumor elimination and, in some cases, facilitating immune evasion through immunosuppressive elements such as regulatory T cells (Tregs), tumor-associated macrophages (TAMs), and myeloid-derived suppressor cells (MDSCs). Additionally, stromal components, including cancer-associated fibroblasts (CAFs), actively contribute to tumor progression by remodeling the extracellular matrix (ECM), promoting angiogenesis, and modulating immune responses. Vascularization is another critical aspect, as abnormal tumor vasculature can create hypoxic regions that drive treatment resistance and tumor aggressiveness. These factors collectively shape the TME and influence therapeutic response, highlighting the need for further research to develop more effective combination therapies targeting both cellular and mechanical components of the TME [20].

Targeting the mechanical properties of the tumor microenvironment (TME) represents an emerging strategy in cancer therapy, particularly in head and neck squamous cell carcinoma (HNSCC) and other solid tumors. Table 2 highlights several clinical trials investigating agents that modulate integrins, stromal components, and extracellular matrix (ECM) stiffness to enhance treatment efficacy. Translating therapies that target the mechanical properties of the TME into clinical practice remains a challenge due to the complex and dynamic nature of tumor–stroma interactions. While preclinical studies have demonstrated the potential of targeting integrins and stromal components to improve therapeutic efficacy, clinical outcomes have often been disappointing, emphasizing the need for refined approaches.

One of the most studied integrin inhibitors, cilengitide, which targets αvβ3 and αvβ5, was evaluated in squamous cell carcinoma in combination with 5-FU, cisplatin, and cetuximab (NCT00705016). Although preclinical studies suggested that integrin inhibition could enhance chemotherapy sensitivity, clinical trials failed to show significant survival benefits. This outcome underscores the complexity of integrin signaling and suggests that successful integration of these therapies may require better patient stratification and combination with immune-modulating agents. PEGPH20, a hyaluronidase enzyme designed to degrade hyaluronic acid (HA) and reduce ECM stiffness, was investigated in esophageal cancer (NCT03281369). Despite the initial promise, the drug was discontinued due to a lack of efficacy and potential toxicities, highlighting the challenge of targeting ECM components effectively. Similarly, saridegib, a GPCR inhibitor targeting the Hedgehog signaling pathway, was tested in HNSCC in combination with cetuximab (NCT01255800). While the Hedgehog pathway is known to play a role in ECM remodeling and stromal cell activation, clinical translation of GPCR inhibitors remains difficult due to an incomplete understanding of their effects on tumor–stromal interactions. These challenges suggest that future efforts in targeting the mechanical properties of the TME will require biomarker-driven patient selection and optimized combination strategies to improve therapeutic outcomes [21].

### 2.1. Types of 3D Models for Head and Neck Cancer

**Figure 2:**
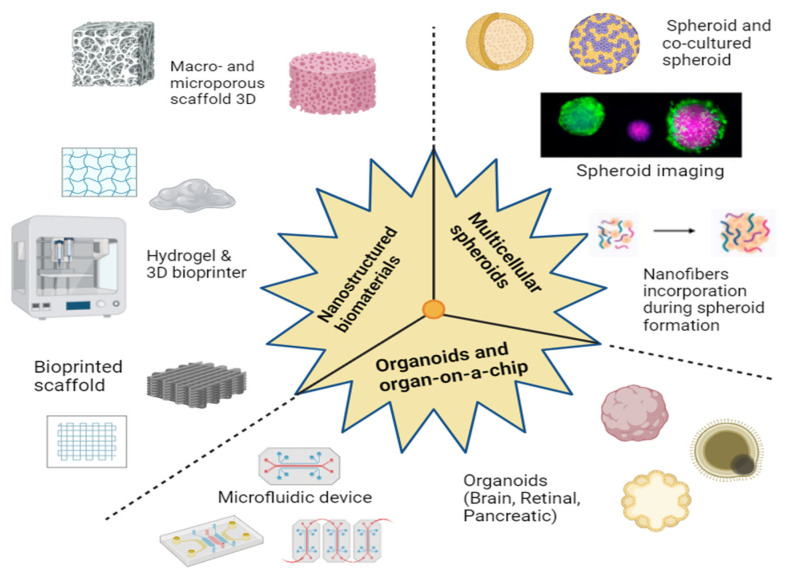
Schematic representation of the most promising technologies and tools for the engineering of 3D in vitro models. Figure created with BioRender (https://www.biorender.com/ (accessed on 16 February 2025) Shiz Aoki, Toronto, Canada).

Three-dimensional cultivation involves the following techniques: growing cellular spheroids and organoids, utilizing bioink, sowing on scaffolds, and decellularized tissues, microfluidics, “organs-on-a-chip”, and 3D bioprinting (Figure 2).

Choosing one of these approaches depends on many factors, including the aim, experimental time frame, and material contents. At present, three-dimensional cell systems are most developed using technologies based on cell spheroids, scaffolds, and hydrogels. These methods are favored due to their affordability, simplicity, scalability, and suitability for high-throughput analysis, making them particularly valuable for drug testing [22].

#### 2.1.1. Spheroid Models

Multi-cellular spheroids derived from tumor cells are among the simplest and most reproducible in vitro tumor models that can accurately reflect how tumor tissue responds to therapy. Spheroids exhibit cellular zoning, which becomes more pronounced as their size increases. When spheroids reach a diameter of around 500 μm, they develop a hypoxic zone and areas of necrosis. Histological examination of large spheroids reveals several distinct layers: an external layer of rapidly proliferating cells, a central layer of senescent or quiescent cells, and an inner layer of necrotic cells. These diverse layers arise due to the restricted diffusion of oxygen and nutrients across the multi-cellular structure [1,23].

**Figure 3:**
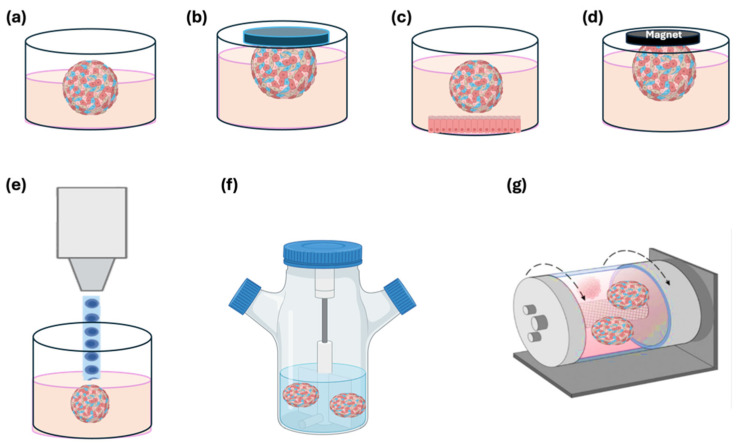
Different methods of cell spheroid method. (**a**) the use of a low-adhesive curved surface, (**b**) the hanging drop method, (**c**) the use of microstructured substrates, (**d**) magnetic levitation using magnetic culture, (**e**) bioprinting, (**f**) cultivation of suspension culture in stirred bioreactors, and (**g**) in spinner flasks.

Spheroid formation occurs naturally when cell–cell interactions outweigh interactions between cells and the culture surface. As a result, all primary techniques for spheroid formation are designed to foster these conditions. These methods involve hanging drop technique, growing cells on ultra-low-adhesion surfaces in dishes or vials, and using rotational systems or magnetic fields. In each of these methods (Figure 3), the key objective is to minimize contact between cells and the culture substrate. Primary cultures are inherently heterogeneous and thus provide more relevant models for personalized medicine. To better replicate the different compositions of tumor tissue, heterospheroids (also known as heterotypic or co-culture spheroids) are used, consisting of tumor cells associated with other cells found in vivo, such as immune cells, fibroblasts, or endothelial cells [1,24].

In many respects, spheroids closely resemble tumor tissue, making them a relevant preclinical model for assessing the efficacy and toxicity of a wide range of anticancer drugs. However, they often fail to properly include intratumoral heterogeneity and the biomechanical and biochemical cues provided by surrounding tissues due to the lack of other supporting cell types and the extracellular matrix that contribute to the tumor microenvironment [22].

Kadlets et al. have developed stable protocols for HNSCC spheroid formation and highlighted their distinct characteristics in comparison to traditional 2D cell culture techniques using FaDu, CAL27, and SCC25 head and neck squamous cell carcinoma cells [25]. The research analyzed the expression of key HNSCC proteins in spheroids and compared the findings with a 2D cell model. Although derived from the same cell line, the spheroids and monolayer cultures exhibited variations in protein expression intensity and staining patterns. Also, Melissaridou et al., in their research, confirmed how the 3D cell cultures (five HNSCC cell lines, namely LK0858B, LK0902, LK0917, LK1108, and LK1122 were used) imitate the in vivo behavior of neoplastic cells within the tumor, suggesting that a 3D culture model is superior to 2D monolayers in the search for new therapeutic targets [26].

To recreate a tumor microenvironment (TME) closer to real conditions, Francois et al. developed a 3D human heterotypic model consisting of head and neck squamous cell carcinoma (HNSCC) cells and different subtypes of macrophages to replicate the interactions between immune cells and cancer cells [27]. The goal is to develop a model for screening immunomodulatory nanomedicines designed to target tumor-associated macrophages (TAMs) in solid head and neck tumors, either as standalone treatments or in combination with standard therapies. A summary of studies comparing 2D and 3D spheroid models is provided in Table 3A.

#### 2.1.2. Organoid Models

**Figure 4:**
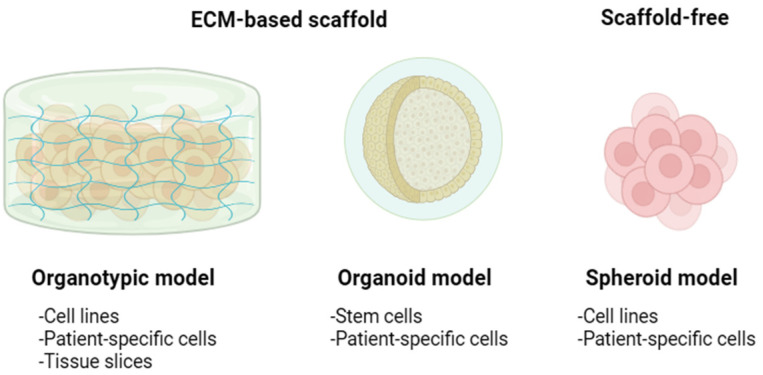
An illustration of organotypic culture models. Organotypic models offer cell–cell and ECM interactions within a 3D culture system. These models involve embedding disaggregated cells or tissues in ECM-based scaffolds. Specifically, organoids are a subtype of organotypic models derived from stem cells or patient-specific cells. In contrast, spheroids can be formed using either single or multiple standard cell lines or patient-derived cells, with or without the inclusion of ECM-based scaffolds. Figure created with BioRender (https://www.biorender.com/, 2025, Shiz Aoki, Toronto, Canada) and adapted from Moya-Garcia et al. [31].

The development of organoid technology, which merges the benefits of spheroids (cell self-organization in 3D) and tissue engineering models (presence of extracellular matrix), has significantly advanced research into the interactions between tumor cells and ECM (Figure 4). As a result, organoids exhibit high genetic and phenotypic similarity to native tissues, preserving the original intratumoral heterogeneity. Organoids used for modeling HNSCC are typically derived from the resected tumor tissue of patients. In recent years, organoids have also been successfully generated from normal and tumor tissues of various organs, including the brain, lungs, esophagus, stomach, intestines, liver, pancreas, kidneys, and salivary glands, among others [1,28].

The number of studies successfully producing HNSCC organoids is limited due to logistical challenges (minimizing the time from tumor tissue resection to delivery to the laboratory), often insufficient volume of biologic material, and stringent requirements for both the qualitative and quantitative composition of the culture medium. Moreover, the efficiency of generating organoids from head and neck tumor tissue rarely exceeds 60–70%, even in well-established laboratories. The primary factor affecting this efficiency is believed to be the quality of the initial biological material, as necrotic tumor tissue has a low organoid-forming capacity [1,29].

Creating organoids requires significant material and time investments, specialized expertise, and close collaboration between clinicians and cell biology researchers. The result is a tumor model that can be maintained in vitro for extended periods, preserving genetic stability while closely resembling the original tissue’s morphology. Organoids are considered valuable in vitro tumor models because they retain a unique set of biomarkers from the donor tissue. Growing evidence indicates that organoids can predict the treatment response of the tumors from which they are derived. Numerous studies highlight the prognostic potential of organoids in predicting how solid tumors will respond to radiotherapy and its combination with other treatments. Furthermore, organoids can be cultured and cryopreserved, facilitating the creation of organoid biobanks containing various cancer subtypes from multiple patients, making them an essential resource for preclinical research [1,30].

Some studies comparing 2D and 3D organoid models are summarized in Table 3B.

#### 2.1.3. Scaffold-Based Models

To enhance the relevance of in vitro tumor models, tissue engineering approaches, such as cell culture on 3D scaffolds, have been utilized. The first HNSCC model utilizing a synthetic scaffold was introduced in 2007. This model involved culturing oral squamous cell carcinoma (OSCC-3) cells within a highly porous poly(lactide-co-glycolide) (PLG) scaffold.

Scaffolds are three-dimensional structures derived from various sources, characterized by parameters such as porosity, mechanical properties, and surface chemistry. They are engineered to replicate the extracellular matrix, offering structural support to cells while encouraging adhesion, proliferation, and differentiation. Natural materials like alginic acid, chitosan, collagen, fibronectin, and hyaluronic acid are preferred over synthetic ones due to their closer resemblance to the human body. In contrast, synthetic polymers (e.g., poly-ε-caprolactone (PCL), polyvinyl alcohol (PVA)) are used in the manufacture of scaffolds to provide better mechanical strength, processability, and more controlled degradation rates. Scaffolds of natural and synthetic origin are often used to create biomimetic tumors and subsequently used in anticancer drug trials. Thus, the use of polylactide scaffolds may help mimic the tumor microenvironment [22,32].

Furthermore, in the field of scaffolds, developments are underway to realize hybrid scaffolds that study the correlation between mechanical and surface chemical properties on the one hand and cellular responses on the other. Understanding these interactions will help to achieve maximum biocompatibility, high cell proliferation, and differentiation when implanting materials at the site of injury. Further advancement and optimization of scaffold-based HNSCC models, considering essential factors such as material cytotoxicity, carcinogenicity, and others, could be beneficial for studying various cancer-related processes. Additionally, these models could help identify similar materials suitable for use as grafts in severe cancer lesions [22,33].

HNSCC has been studied with models using various scaffolds including sandwich-like collagen scaffolds [34], microtiter plates [35], decellularized tongue extracellular matrix [36], poly(L-lactide-co-ε-caprolactone) (PLCL) scaffolds [37], and 3D-printed scaffolds based on β-tricalcium phosphate [38]. Thus, scaffold-based models provide for co-culture with various cell types, considering the physicochemical properties of the extracellular matrix (as in hydrogel-based models) and the influence of microstructure/topography/curvature on tumor 3D growth. For example, these models could focus on testing diagnostic agents to detect cancer markers related to cell adhesion or chemotherapeutic agents targeting the cytoskeleton [22,39].

#### 2.1.4. Bioprinted and Hydrogel-Based Models

In recent years, 3D tissue printing has advanced significantly. This technique involves building three-dimensional structures by precisely layering bioink, which contains cells, cytokines, and extracellular matrix components. Bioink typically consists of a biocompatible hydrogel embedded with single cells or cell spheroids [22,40]. These techniques differ considerably in various factors, such as equipment and material costs, printing speed, resolution, limitations on the maximum size of the printed object, and the types of hydrogels that can be used. At present, there are only a few studies detailing the successful application of bioprinting methods for modeling HNSCC. Kort-Mascort et al. showed that their 3D bioprinted model of HNSCC effectively serves as an advanced tissue-engineered model. In this approach, a hydrogel scaffold is employed, and the printing method allows precise control over tissue architecture, surpassing traditional gel layering techniques. However, to date, bioprinted HNSCC models have been developed primarily using immortalized cell lines, primarily due to the high cell density required for achieving the desired outcomes [41].

A key goal for researchers is to create 3D models using patient-derived HNSCC cells to better replicate biological features and potentially advance personalized medicine applications. A promising strategy involves utilizing synthetic extracellular matrices that replicate the characteristics of natural extracellular matrices found in vivo. These synthetic matrices can be composed of natural polymers, such as alginate, chitosan, and pectin, or chemically synthesized polymers like PEG and PVA. The primary requirements for these hydrogels include biocompatibility and suitable physicochemical properties. Additionally, their characteristics—particularly mechanical properties such as density, stiffness, viscoelasticity, and plasticity—can be precisely adjusted and regulated through chemical or biotechnological synthesis. Compared to natural matrices, synthetic matrices offer greater control over the simulation of the tumor cell microenvironment, enabling more accurate modeling of in vivo conditions. The mechanical properties of hydrogels affect tumor cell proliferation, migration, and cell function formation through cell surface adhesion proteins, linker proteins, and cytoskeletal networks, and stimulate signaling for spheroid and organoid development [42].

Thus, the ability to modulate properties allows for the specific setting and selection of specific types of tumor cultures with different morphology and cellular behavior concerning HNSCC. As a result, hydrogel-based models can be used to test anticancer drugs in terms of penetration into tumor cells via the extracellular matrix.

#### 2.1.5. Tumor Explants and Histocultures

Another 3D cell model is the patient-derived tumor explant (PDE) culture, where a tissue fragment is maintained alive under ex vivo conditions. This model involves minimal manipulation: the tissue is mechanically fragmented into pieces approximately 1–3 mm^3^ and then placed in a culture medium. This approach maintains the different cellular and extracellular components of the original tumor, including both the extracellular matrix and associated tumor cells. Despite its advantages in maintaining tumor tissue architecture and ease of production, the use of a PDE as an in vitro model is limited by its fragility. Typically, PDEs are employed for rapid evaluation of drug efficacy and/or toxicity within 5–7 days post-isolation [1,43]. It has been observed that the viability of HNSCC explants declines from 90% to 30% within a week of cultivation.

An alternative approach for this type of 3D model is histoculture. In histoculture, tumor tissue is mechanically processed not with surgical instruments but using a vibratome, which produces thick, unfixed tissue sections. Similar to explants, these sections preserve the tumor cells within their native microenvironment, including the extracellular matrix, immune cells, and stromal components. As a result, explants and histocultures of HNSCC are relatively simple to obtain and efficient for quickly evaluating tumor tissue responses to drug treatments, demonstrating their potential for use in personalized medicine applications [44].

#### 2.1.6. Microfluidics-Based Models (Tumor-on-a-Chip)

Tumor-on-a-chip is a technique that has seen significant development in the past decade, allowing for precise manipulation of small liquid volumes and control over nutrient concentrations in both space and time. This is achieved through microchannels (ranging from 1–1000 µm in cross-section) where the liquid flow remains laminar. In addition, microfluidics has the advantage of being able to handle small amounts of experimental samples and reagents. Microfluidic technology is particularly useful in studying oncological diseases due to the correlation between cancer cell migration and the gradient of various chemokine concentrations. Notable recent developments in microfluidics include 3D microfluidic chips and microfluidic devices for managing chemotactic migration of HNSCC cells. Furthermore, it is easier to use and enables research of HNSCC that closely mimics human physiology compared to animal testing [45].

Therefore, tumor-on-a-chip technology represents a valuable tool for personalized evaluation of the efficacy of various antitumor agents, either individually or in combination. It allows for dynamic monitoring of how tissue samples from HNSCC patients respond to different treatments. The progress in microfluidic platforms holds the potential to improve patient outcomes by enabling the selection of the most effective personalized treatment strategies. Some studies comparing 2D and 3D microfluidic device models are summarized in Table 3C.

**Table 3:** Existing models of head and neck cancers (HNCs) have been reported in literature.

Models	2D vs. 3D Geometry	Primary vs. Cell Lines	Drugs Simulant	Aim	Authors and Ref.
(**A**) Spheroids	2D: Monolayer control 3D: Forced floating and hanging drop methods	Primary: Tumor biopsy from HNSCC Cell lines: CAL-27, FaDu	Cisplatin, 5-FU, 2-Gy radiation	To compare 2D and 3D methods as platforms for testing chemotherapy and radiotherapy	Hagemann et al. [46]
	2D: Not studied 3D: Forced floating method	Primary: Patient-derived cancer-associated fibroblasts Cell lines: LK0902, LK0917	All-trans retinoic acid (ATRA)	To evaluate a 3D model as a therapy testing platform	Gorican et al. [47]
	2D: Not studied 3D: Forced floating method	Primary: Patient-derived cancer-associated fibroblasts Cell lines: LK0902, LK0917	Cisplatin, cetuximab	To evaluate a 3D model as a platform for testing chemotherapy and immunotherapy	Magan et al. [48]
(**B**) Organotypic	2D: Monolayer control 3D: The forced floating method was employed initially, followed by the transfer of the cells into Matrigel^®^	Primary: Tumor biopsy from H&N squamous cell carcinoma Cell lines: MDA-HN2016-2, MDA-HN2016-18, MDA-2016-21	Cisplatin, docetaxel	To compare 2D and 3D methods as platforms for evaluating chemotherapy and sensitivity	Tanaka et al. [49]
	2D: Monolayer control 3D: Decellularized extracellular matrix from tongue tissue, sourced from mice and pigs alongside a Matrigel^®^ matrix	Primary: Tongue squamous cell carcinoma and cancer-associated fibroblasts derived from patients Cell lines: CAL-27	Cisplatin	To compare 2D and 3D methods as platforms for chemotherapy screening and regenerative applications	Zhao et al. [50]
	2D: Dynamic flow 3D: Collagen hydrogel adhesion chamber, dynamic flow, syringe pump	Primary: Human tubular lymphatic vessels and fibroblasts associated with cancer Cell lines: HLEC2500, HorF2640	Not studied	A dynamic culture technique employed as a platform for studying angiogenesis	Burghartz et al. [51]
(**C**) Microfluidic Devices	2D: Unexposed control, dynamic flow 3D: Tissue chamber, dynamic flow, syringe pump	Primary: Human thyroid tissue samples Cell lines: Not studied	Etoposide (topoisomerase II inhibitor), SP600125 (JNK inhibitor)	A dynamic culture method, used as drug screening platform	Riley et al. [52]
	2D: Unexposed control, dynamic flow 3D: Biopsy chamber, dynamic flow, syringe pump	Primary: Staging of human biopsies of laryngeal, oropharyngeal, and oral cavity tumors at T2 through T4 Cell lines: Not studied	Not studied	A dynamic culture method, used as maintenance platform	Bower et al. [53]
	2D: Dynamic flow 3D: Collagen hydrogel adhesion chamber, dynamic flow, syringe pump	Primary: Human tubular lymphatic vessels and fibroblasts associated with cancer Cell lines: HLEC2500, HorF2640	Not studied	A dynamic culture method, used as an angiogenesis platform	Lugo-Cintrón et al. [54]

## 3. Experimentation with 3D Models of HNSCC

### 3.1. Thyroid Gland

In a study conducted by Peng Su, Chao Yue et al. [12], uniaxial compression tests were conducted on fresh porcine thyroid tissue at quasi-static strain rates of 0.005 s^−^¹ and 0.05 s^−^¹, with loading applied both perpendicular and parallel to the thyroid surface. These experiments were conducted using a biomechanical testing platform equipped with a Nano25 high-precision sensor to evaluate how strain rate and loading direction influence the mechanical properties of porcine thyroid tissue. The findings revealed that porcine thyroid tissue showed minimal strain rate sensitivity within the low strain rate range of 0.005 s^−^¹ to 0.05 s^−^¹. Furthermore, the loading direction had little effect on the tissue’s mechanical properties, indicating that the tissue behaves as a homogeneous material. The mechanical characteristics of porcine thyroid tissue were also examined under quasi-static conditions. It was found that Young’s modulus showed no significant variation across different strain rates and loading directions. The average Young’s modulus was found to be approximately 2.233 × 10^−5^ MPa for the small deformations and 3.108 × 10^−3^ MPa for the large deformations. To analyze the mechanical properties of porcine thyroid tissue under quasi-static conditions, it was modeled as an incompressible, isotropic superelastic material. A hyperelastic constitutive model was developed using the Yeoh strain energy density function to describe the tissue behavior at a low strain rate. The three parameters of the Yeoh function were determined through fitting and were found to be: C_10_ = 1.9 × 10^−3^ MPa, C_20_ = −2.3 × 10^−3^ MPa, C_30_ = 0.04 MPa.

**Figure 5:**
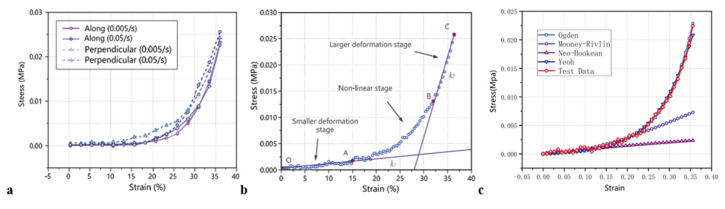
(**a**) The stress–strain curve for the quasi-static compression test; (**b**) Analysis of the mechanical properties of porcine thyroid gland tissue; (**c**) Fitting of the thyroid stress–strain curve to the constitutional model. (Reprinted with permission from Ref. [12]).

The stress–strain curves (Figure 5) derived from the simulation were generally consistent with the experimental data. However, some discrepancies were observed, likely due to factors that could not be fully replicated in the actual tests, such as localized deformations and uneven stress distributions. The quasi-static mechanical properties of the thyroid, obtained from uniaxial unconfined compression tests and represented by the developed hyperelastic constitutive model, are highly suitable for digital modeling of thyroid tissues. These findings are presented in Table 4A.

### 3.2. Head and Neck Cancer (HN-5) Cell Line

**Figure 6:**
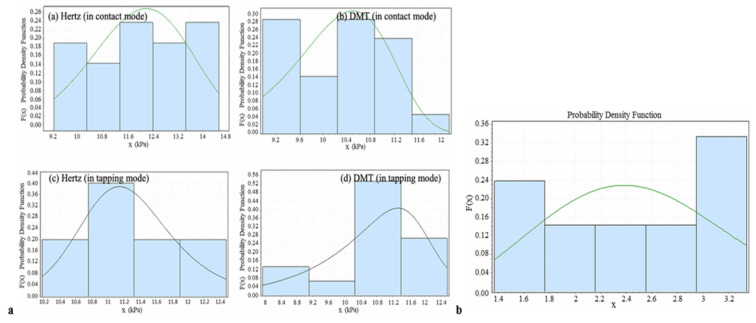
**Panel a**: (**a**) Normal distribution and histogram of Young’s modulus calculation in contact mode with the Hertz model.; (**b**) DMT contact model results in contact mode; (**c**) Hertz contact model results in tapping mode; and (**d**) DMT contact model results in tapping mode; **Panel b**: The histogram displays the extracted values for the adhesion force measured in contact mode. (Reprinted with permission from Ref. [55]).

In a paper by M.H. Korayem et al. [55], experimental methodologies utilizing atomic force microscopy (AFM) were employed to investigate the physical and mechanical properties of the head and neck carcinoma (HN-5) cell line. Young’s modulus was calculated by conducting repeated indentations at a minimum of 10 different points and utilizing two contact modes. The results showed that regions with lower elevation had a higher elasticity modulus, and vice versa. The average elasticity modulus for the cells was found to be 12 kPa. Furthermore, the adhesion force, which plays a key role in friction and manipulation, was experimentally measured. Similar to the elasticity modulus, maximum adhesion was recorded at the thinner edges of the cells, while the minimum adhesion was observed at the center of the cells. As adhesion increased, more force was required to detach the cells from the surface, leading to a corresponding increase in the manipulation force. In air medium and contact mode of the cantilever, the average adhesion value was recorded as 2.47 nN (Figure 6).

**Figure 7:**
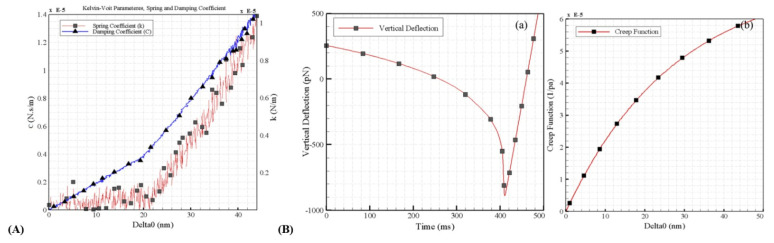
**Panel A**: Kelvin–Voigt components (spring constant and damping coefficient) of the HN-5 cell; **Panel B**: (**a**) Vertical deflection over time; (**b**) Creep function as a function of the oscillation amplitude of indentation. (Reprinted with permission from Ref. [55]).

Another characteristic measured was the folding factor of the cell, which was assessed using two different methods, resulting in values of 1.19 and 1.40, respectively. It was concluded that both the spring constant and the damping coefficient are directly affected by the indentation depth. Following this, the creep function for a cell was derived, simulated using a basic viscoelastic model, such as the Kelvin–Voigt model (Figure 7):

In viscoelastic materials, the creep–compliance curve shows that the compliance (strain) of the material increases with time under constant stress. The region of interest in the creep test corresponds to the portion of delayed elastic strain. This implies that if the stress is removed, the sample will gradually return to its original state over time t. The results are presented in Table 4B.

### 3.3. Cervical Lymph Nodes

**Figure 8:**
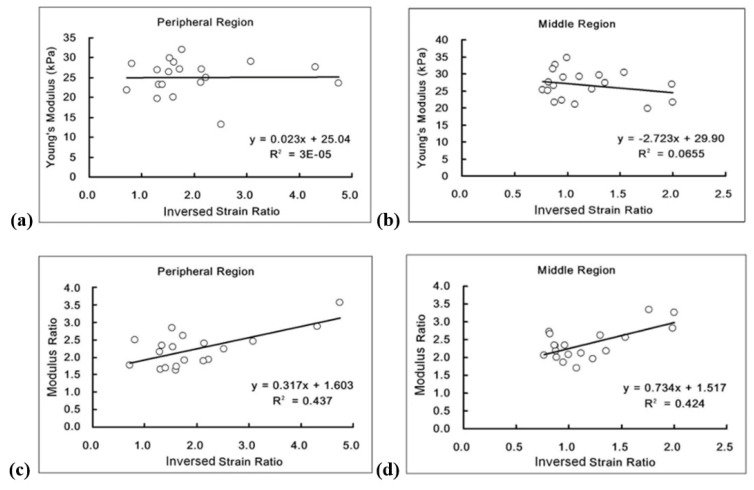
Correlation between Young’s modulus/elastic modulus ratio and inverse strain ratio for peripheral and intermediate lymph nodes. (**a**,**b**) pertain to the results of Young’s modulus measurements, while (**c**,**d**) relate to the modulus ratio findings. (Reprinted with permission from Ref. [56]).

Cervical lymph nodes are commonly involved in metastatic head and neck cancers. Metastatic lymph nodes are usually firm, while benign nodes are softer. In clinical practice, palpation is still a standard technique used by ear, nose, and throat (ENT) specialists and oncologists to assess cervical lymphadenopathy, despite the inherent subjectivity of this method. The study conducted by Queeny Wing-Han Yuen et al. [56] aimed to provide a quantitative assessment of lymph node stiffness by measuring the tissue stiffness of pig lymph nodes using two methods: elastography for strain measurement of the entire lymph node and indentation for modulus determination of sliced lymph node specimens. The entire lymph nodes were embedded in agar–gelatin blocks for elastography, while only sliced specimens were used for indentation testing. No significant correlation was found between Young’s modulus and the inverse strain ratio in both the peripheral and middle regions. This discrepancy may be due to the fact that the inverse strain ratio indicates the stiffness contrast between the lymph node and the phantom material, while Young’s modulus reflects the absolute stiffness of the lymphatic tissue. When the modulus ratio was applied, a moderate correlation was observed between the two methods, likely influenced by factors such as elastography image quality, interfacial bonding, boundary conditions, tissue geometry, and size. The investigation revealed that the pig lymph nodes had distinct smooth boundaries, easily separated from surrounding connective tissues. However, some connective tissues remained attached to the lymph nodes when embedded in agar–gelatin blocks, potentially increasing the surface area for bonding with the phantom material. Elastography revealed that the stiffness of the middle region of the lymph nodes was like that of the background material, while the peripheral region was harder than the middle region. Conversely, the indentation test revealed that the central region was stiffer than the outer region. This discrepancy may be due to the direction of compression in elastography (parallel to the slice surface) versus indentation (perpendicular to the slice) (Figure 8).

Additionally, although strain measurements were taken at various sites of the lymph node to assess strain distribution, these did not perfectly align with the plane used for modulus measurements. The indentation system demonstrated satisfactory repeatability, with a prestudy evaluation on 12 silicone samples of varying stiffness showing an intraclass correlation coefficient (ICC) of 0.99, indicating a repeatability of 99%, and a repeatability coefficient (according to Bland et al. [57]) of 10.7 kPa. The observed difference in Young’s modulus between the surrounding background material (11.78 ± 2.80 kPa) and the lymph node tissue (26.06 ± 6.03 kPa) can be attributed to the inherent stiffness differences, as indicated by this coefficient. Due to equipment constraints, the sliced specimens were relatively thick, exceeding 7 mm. Sample thickness is an important consideration, especially for pigs, as their lymph nodes may contain multiple nodelets, leading to significant internal tissue heterogeneity. Thinner slices are preferred, as they generally display more consistent properties throughout the tissue depth. In this study, a cutting technique was developed to create slice specimens with uniform thickness across a larger cross-sectional area (8–10 cm in width and length). Thicker sliced specimens were favored for the indentation test due to their superior quality compared to thinner slices. The study was conducted solely on normal lymph nodes obtained from market pigs assumed to be healthy. The results are presented in Table 4C. In conclusion, this study aimed to assess the stiffness of porcine lymph nodes by measuring strain through elastography and Young’s modulus using a push-in test. Both methods highlighted significant differences in parameters between the peripheral and middle regions of the lymph node, with the results from the two tests showing a moderate correlation [56].

**Table 4:** Summary of mechanical properties extracted from the models in literature. (**A**) Young’s modulus in the small (E_1_) and large (E_2_) deformation phase of the porcine thyroid (**a**); Yeoh hyperelasticity constitutive model evaluated by material parameters (C_10_, C_20_, C_30_) and correlation coefficient (R^2^) (**b**); Statistics on stress in stress–relaxation tests (**c**). (**B**) Young’s modulus in contact and tapping modes for Hertz and DMT contact models (**a**); adhesion strength of HN-5 cells (**b**); folding factor of HN-5 cells based on surface method (**c**). (**C**) Results of Elastography and Indentation tests (mean ± SD).

Type of Model	Mechanical Features	Type of Value	Value	Ref.
(**A**) Thyroid Gland	(**a**) Young’s modulus	Mean	E_1_ (MPa) = 2.333 × 10^−5^ E_2_ (MPa) = 3.108 × 10^−3^	[12]
	(**b**) Hyperelasticity constitutive model	Yeoh	C_10_/MPa = 1.9 × 10^−3^ C_20_/MPa = −2.3 × 10^−3^ C_30_/MPa = 0.04 R^2^ = 0.999	
	(**c**) Stress–relaxation tests	Mean	Initial stress (MPa) = 2.433 × 10^−2^ Last stress (MPa) = 3.693 ×10^−3^	
(**B**) Head and Neck Cancer (HN-5) Cell Line	(**a**) Young’s modulus (kPa)	Mean	Contact/Hertz = 11.95 Contact/DMT = 10.29 Tapping/Hertz = 11.28	[55]
	(**b**) The adhesion force	Mean	2.47 nN	
	(**c**) Folding-surface method	-	Project area (nm^2^) = 1873.02 Surface area (nm^2^) = 2628.13 Folding factor (first method) = 1.19 Folding factor (second method) = 1.403	
(**C**) Cervical Lymph Nodes	Elastography strain ratio	-	Peripheral region = 0.61 ± 0.32 * Middle region = 0.93 ± 0.33	[56]
	Indentation Young’s modulus (kPa)	-	Peripheral region = 25.39 ± 6.14 * Middle region = 26.73 ± 5.64	
	Indentation modulus ratio	-	Peripheral region = 2.21 ± 0.60 Middle region = 2.33 ± 0.51	

* Indicates a significant difference (*p* < 0.05).

### 3.4. Laryngotracheal Cartilage

In a review written by Christine M. Pauken et al. [58], the literature on the mechanical, cellular, and proteomic properties of laryngotracheal cartilages was comprehensively examined to establish a foundational understanding. A summary of their findings is provided in Table 5.

The concepts outlined hold significant implications for laryngotracheal cartilage tissue engineering. Currently, there is limited understanding of the mechanical properties of these cartilages. Tissue stiffness, both under tensile and compressive conditions, is a key factor, especially considering that the stiffness of thyroid and arytenoid cartilages can affect vocal performance, particularly in aging tissues where ossification may alter their stiffness. Furthermore, the cricoid and tracheal cartilages need to maintain sufficient stiffness to keep the airway open. Unfortunately, the compressive and tensile properties of human cricoid, tracheal, arytenoid, and epiglottic cartilages remain largely unexplored. Other important material properties, such as bending modulus and joint lubrication, are also critical for cartilage function. The bending modulus at various points on the epiglottis is essential for its proper function, while effective lubrication is crucial for the movement of the cricoarytenoid and cricothyroid joints. Testing these cartilages should be linked to factors like cell density, glycosaminoglycan (GAG) concentration, the distribution of key proteins, collagen and elastin networks, and areas of ossification. A thorough understanding of these factors will greatly enhance the ability of professionals in regenerative medicine to reconstruct the laryngotracheal system [58].

## 4. Comparison of In Vitro and In Vivo Models in HNSCC Research

The development of reliable models that closely replicate the tumor microenvironment (TME) is crucial for advancing cancer research and enhancing personalized medicine. While in vitro and in vivo models each have their own strengths and weaknesses, their integration is essential for achieving translational success. Recent advancements have improved the capacity of in vitro systems to mimic key features of in vivo conditions, adhering to the principles of the 3Rs (replacement, reduction, and refinement) to minimize the use of animal models.

In vitro models, such as 3D cultures, organoids, and bioprinting techniques, provide a controlled environment to study tumor biology, offering insights into drug responses, cell–cell interactions, and stromal remodeling. In Table 6 the main features of the most common 3D models used for HNSCC are compared.

For instance, Wafa Wahbi et al. [60] demonstrated the utility of Myogel-coated wells and 3D microfluidic chips in replicating the extracellular matrix (ECM) and tumor–immune interactions. These models facilitate the testing of patient-derived cells, enabling more precise development of personalized therapies. However, the lack of systemic factors, including angiogenesis and immune responses, represents a key limitation of in vitro approaches. Conversely, in vivo models, such as zebrafish xenografts, provide a more comprehensive representation of the tumor microenvironment, incorporating systemic factors like immune cell dynamics and vascularization. Zebrafish assays have shown a high concordance with clinical outcomes, particularly in testing chemotherapeutic, radiotherapeutic, and targeted therapies. By integrating these complementary models, researchers can enhance the physiological relevance of preclinical studies, paving the way for more effective, patient-specific therapeutic strategies.

On the other hand, in vivo models, such as murine xenografts and zebrafish larvae, analyzed in an article written by Roosa Hujanen et al. [64], maintain the heterogeneity of primary tumors and enable the study of systemic interactions, including metastasis and vascular mimicry (VM). Zebrafish models have shown a high correlation with clinical outcomes, making them a cost-effective and ethical alternative to traditional mammalian models. For instance, studies in oral squamous cell carcinoma (OSCC) demonstrated a 77% alignment between zebrafish xenograft results and patient responses, underscoring the reliability of these models.

Despite these differences, both in vitro and in vivo systems share the goal of faithfully reproducing tumor behaviors to facilitate drug discovery and improve therapeutic strategies. The increasing correspondence between in vitro and in vivo findings highlights the potential for advanced in vitro models to complement or even replace certain in vivo approaches. By integrating in vitro and simplified in vivo models, researchers can better simulate the complexities of the TME while adhering to ethical research practices.

In conclusion, the synergy between in vitro and in vivo systems not only enhances our understanding of tumor biology but also provides a pathway toward reducing animal use in research. The continued refinement of these models will bridge the gap between preclinical findings and clinical applications, accelerating the development of effective and ethical cancer therapies [65].

## 5. Discussion and Future Prospects

Head and neck cancers comprise a diverse range of malignancies that arise from several anatomical areas, including the oral cavity, pharynx, larynx, nasal cavity, and salivary glands. Precisely identifying and characterizing cancerous tissues is crucial for accurate diagnosis, treatment strategy development, and enhancing patient outcomes. Three-dimensional (3D) in vitro models have emerged as promising tool to enhance our understanding and prediction of HNSCC tissue behavior, offering more physiologically relevant conditions that closely mimic the mechanical and structural properties of native tumor tissues compared to traditional two-dimensional (2D) cultures [66].

This review highlighted the critical role of mechanical properties in these 3D models and their characterization, emphasizing their importance in advancing HNSCC in vitro scientific research. Mechanical properties such as stiffness, elasticity, and dynamic behavior are crucial in replicating the native tumor microenvironment. For example, the stiffness of the extracellular matrix (ECM) influences cancer cell behavior, including proliferation, migration, and response to therapy. In HNSCCs, the tumor microenvironment is characterized by a heterogeneous ECM that affects tumor growth and metastatic potential. Three-dimensional models that accurately mimic these mechanical properties enable a more accurate study of tumor biology and the development of effective therapeutic strategies [67].

Various 3D culture techniques, including spheroids, organoids, hydrogels, and bioprinted tissues, have been developed to replicate the complex architecture and mechanical properties of HNC tissues. Each 3D model technique offers unique advantages and limitations in mimicking the head and neck cancer environment. For example, spheroids are easy to use and suitable for high-throughput screening but lack the complexity of native tumors. Organoids, while providing patient-specific models with greater complexity, are more expensive and require technical expertise. Three-dimensionally bioprinted models offer precise control over the spatial arrangement of cells and ECM components, making them powerful tools for studying cell–cell and cell–matrix interactions, drug responses, and tissue mechanics. However, bioprinting is technically complex and expensive, limiting its accessibility for large-scale studies. Hydrogels and ECM-mimicking scaffolds provide a supportive and customizable environment for cell growth, closely resembling the biochemical and mechanical properties of the native ECM. While highly biocompatible, their design and fabrication remain technically challenging, and the cost of materials is often high, restricting their use to specialized applications [68].

Despite the significant progress made, several challenges must be addressed to develop realistic 3D in vitro models for HNSCC research. The lack of standardized protocols affects reproducibility and comparability across studies. Current models often fail to fully capture the complexity of the tumor microenvironment, including immune system interactions, vascularization, and cross-talk between different cell types. Integrating multiple 3D models may provide a more dynamic and comprehensive system that better replicates the physiological tumor environment [69].

To further advance the field of 3D in vitro modeling for HNSCC, the integration of artificial intelligence (AI) tools, such as deep learning (DL) and machine learning (ML), represents a promising approach. These computational technologies have the potential to address some of the key limitations of 3D tumor models by improving model standardization, automating data analysis, and optimizing experimental design. Given the increasing importance of AI-driven tools in biomedical research, their role in refining 3D models should be explored in depth. Moreover, the lack of longitudinal studies and high-throughput applications in TME research has significantly hindered the clinical translation of therapies targeting mechanical properties [70]. Deep learning (DL) and machine learning (ML) can play a pivotal role in addressing these gaps by enabling real-time data analysis, predictive modeling, and automation of complex biological assessments [71].

For example, one of the key applications of ML in 3D in vitro models is in the automated analysis of organoid morphology and fluorescence-based imaging. Traditional manual assessments are often time-consuming and prone to variability, whereas ML-based tools, such as ML-based Organoid Analysis software (MOrgAna: the software has been tested with Python versions 3.7, 3.8, and 3.9), have demonstrated their ability to analyze structural and functional parameters of organoids with higher precision and reproducibility. These advancements contribute to reducing human bias and increasing the reliability of in vitro tumor models [72].

Beyond ML, deep learning, a subset of ML that uses artificial neural networks to learn, further refines these capabilities by enabling the automatic extraction of relevant features from complex datasets without requiring manual input. For example, the development of self-learning microscopes powered by DL allows for real-time adaptive imaging and automated detection of morphological changes in 3D cultures. This approach can significantly improve the reproducibility of experiments, making it easier to compare results across different laboratories [73].

In addition to improving morphological assessments, DL models hold promise in predicting mechanical properties of 3D constructs. By analyzing large datasets, these systems can identify correlations between matrix composition, stiffness, and tumor behavior, leading to more precise control over model design. Recent studies conducted by JungHo Kong et al. [74] have demonstrated that deep learning can accurately predict the mechanical properties of multi-material metamaterials, significantly reducing the time required for evaluation and enabling large-scale parallel computing. This capability allows for the rational design of structures that exhibit a broad range of elastic properties while meeting additional mechanical constraints, such as stress uniformity to enhance fracture and fatigue resistance. Similarly, in the context of 3D tumor models, these computational approaches can be leveraged to optimize scaffold stiffness, biomaterial composition, and mechanical microenvironment parameters to better mimic in vivo conditions.

Moreover, DL-driven image analysis can facilitate the integration of patient-derived 3D cultures into personalized medicine by assessing drug responses in a high-throughput and automated manner. The ability of DL models to process vast amounts of imaging and mechanical data enhances reproducibility and predictive accuracy, ultimately refining the development of in vitro tumor models. By incorporating mechanical constraints into DL frameworks, researchers can further bridge the gap between experimental and computational modeling, paving the way for more physiologically relevant 3D systems in cancer research and therapeutic development [75].

Despite these advantages, the implementation of DL and ML in 3D in vitro modeling still faces challenges. One of the primary limitations is the need for extensive annotated datasets for proper algorithm training. Additionally, computational demands and hardware requirements can pose barriers for many research laboratories. However, the establishment of standardized imaging databases for 3D cultures could accelerate progress in this field, enhancing model reproducibility and predictive accuracy. By integrating DL and ML approaches, researchers can overcome some of the existing limitations of 3D in vitro models, particularly in standardization, reproducibility, and high-throughput analysis. As these technologies continue to evolve, their application in tumor modeling is expected to refine in vitro systems, bridging the gap between preclinical research and clinical applications.

## 6. Conclusions

The development of 3D tumor models has significantly advanced research in the TME by providing more physiologically relevant platforms than traditional 2D cultures. However, their full potential in high-throughput drug screening and clinical translation is hindered by a lack of standardization in model generation, characterization, and validation. Different 3D culture systems, such as tumor organoids, spheroids, and bioprinted models, exhibit substantial variability in ECM composition, mechanical properties, and tumor–stroma interactions, leading to inconsistencies in experimental outcomes.

A major challenge in using these models is reproducibility, as variations in cell sourcing, ECM stiffness, and culture conditions make it difficult to compare results across studies. Additionally, the absence of standardized methods for measuring mechanical properties and assessing drug responses complicates the translation of TME-targeting therapies from preclinical models to clinical applications. Many 3D models also fail to fully integrate immune and stromal components, limiting their ability to replicate the complex tumor–stroma interactions that influence therapy resistance.

While 3D in vitro models have demonstrated their potential in head and neck squamous cell carcinoma (HNSCC) research, further advancements are needed to enhance their physiological relevance and predictive accuracy. One critical improvement involves implementing longitudinal studies to track tumor progression and treatment response over time, providing insights into resistance mechanisms and therapeutic efficacy. Additionally, integrating genomic, proteomic, and metabolomic data with 3D models could offer a more comprehensive understanding of tumor biology, facilitating the identification of new therapeutic targets. This multi-omics approach aligns with personalized medicine principles, allowing for the development of tailored treatment strategies.

By continuing to refine and integrate 3D model techniques, we can achieve more accurate and comprehensive insights into HNSCC biology. Advancements in standardization, multi-system integration, and high-throughput analysis will further strengthen these models, ultimately leading to more effective and personalized therapeutic strategies. As these technologies evolve, they hold significant potential for improving both preclinical research and clinical outcomes in HNSCC.

## Figures and Tables

**Table 1:** Mechanical properties of distinct human cancer cell types reported in the literature.

Cancer	Normal	Malignant	Ref.
Esophageal	EPC2: E^(1)^ = 4.72 kPa (modal)	CP-A: E = 3.08 kPa (modal) CP-D: E = 2.64 kPa (modal)	[14]
Melanoma	Human epidermal melanocytes, adult (HEMa): E = 14.3 ± 0.8 kPa	VGP (primary tumor): E = 9.91 ± 0.55 kPa Skin metastasis (WM239): E = 7.8 ± 0.61 kPa Lung metastasis (A375P): E = 5.7 ± 0.61 kPa	[15]
Oral squamous cell	OKF-4: δ^(2)^ = 0.69 ± 0.46 × 10^−2^ Pa^−1^ OKF-6: δ = 0.75 ± 0.49 × 10^−2^ Pa^−1^	CAL-33: δ = 1.99 ± 1.63 × 10^−2^ Pa^−1^ CAL-27: δ = 1.59 ± 0.75 × 10^−2^ Pa^−1^ BHY (OSCC): δ = 1.86 ± 0.76 × 10^−2^ Pa^−1^ HNCs: δ = 1.75 ± 0.76 × 10^−2^ Pa^−1^	[16]
Squamous cell carcinoma	Human mammary fibroblasts: E ~1 kPa	SCC38: E ~0.75 kPa	[17]
Thyroid	S748: E ~2211–6879 Pa	S277: E ~1189–1365 Pa	[18]

^(1)^ Young’s modulus (E); ^(2)^ Compliance (δ).

**Table 2:** Clinical trials investigating agents targeting the mechanical properties of the Tumor Microenvironment (TME).

Target	Agent	Cancer Type	Combined with	Clinicaltrial.gov ID
Integrin	Cilengitide	Squamous cell carcinoma	5-FU + cisplatin + cetuximab (EGFR inhibitor) (PFE)	NCT00705016
Stromal components	PEGPH20 (HA) (discontinued)	Esophageal	Leucovorin, cisplatin, paclitaxel, oxaliplatin, fluorouracil, atezolizumab, cobimetinib, ramucirumab, BL-8040, linagliptin, tiragolumab	NCT03281369
Stromal components	Saridegib (GPCR inhibitor)	Head and neck squamous cell carcinoma (HNSCC)	Cetuximab	NCT01255800

**Table 5:** Summary of knowledge on biomechanical properties of Laryngotracheal Cartilage [58].

Structure	Cartilage Type	Biomechanical Properties	Ref.
Thyroid cartilage	Hyaline	Undergoes forces from both intrinsic and extrinsic muscles Increased stiffness due to the process of ossification Interacts with the cricoid through a synovial joint that exhibits three different morphological variants	[59]
Cricoid cartilage	Hyaline	Has two pairs of synovial joints for connecting with the thyroid and arytenoid cartilages A radial expansile force averaging 97.25N is necessary to break human cricoid cartilage A study in horses indicates that the compressive properties of cricoid cartilage vary regionally	[60]
Epiglottic cartilage	Elastic	Parameters for rabbits are characterized by a Poisson’s ratio of 0.250 ± 0.115, permeability of 8.01 ± 5.16 × 10^−16^ m^4^/N·s, and an aggregate modulus of 0.290 ± 0.174 MPa The aggregate modulus of the epiglottis differs considerably from that of other elastic cartilages	[61]
Arytenoid cartilage	Hyaline and elastic	The bulk aggregate modulus for equine tissue is 0.42 ± 0.11 MPa, with a permeability of 1.303 ± 0.191 mm^4^/N·s	[62]
Tracheal cartilage	Hyaline	The average tensile modulus at the abluminal surface is 13.6 ± 1.5 MPa The tensile modulus tends to decrease as depth increases The stiffness of tracheal cartilage increases as age advances Differences in the tensile moduli and the viscosity coefficient of tracheal cartilage influence the subglottal acoustic spectra	[63]

**Table 6:** In vitro models of HNSCC: summary of main features adapted from Arutyunyan et al. [1].

	Spheroids and Heterospheroids	Tissue-Engineered Models	Bioprinted Models	Organoids	Explants and Histocultures
Source	Patient-derived tissues, primary cell cultures, immortalized cell lines	Patient-derived tissues, primary cell cultures, immortalized cell lines	Patient-derived tissues, primary cell cultures, immortalized cell lines	Patient-derived tissues	Patient-derived tissues
Heterogeneity of tumor cellular composition	Depends on the source	Depends on the source	Depends on the source	Preserved	Preserved
ECM	-	Natural/synthetic polymers, decellularized tissue	Bioink based on hydrogels	Basement membrane matrix, collagen	Native
Tissue architecture, pathophysiological gradients	Partially reconstituted	-	Reconstituted	-	Preserved
In vitro culture duration	-	Limited	Limited	-	-
Difficulty of obtaining	Medium	Medium	High	High	Medium
Major advantages	The most commonly available 3D model	The benefit of analyzing ECM–cell interactions lies in the ability to develop a model with precise linear dimensions	Generating synthetic tumor tissue with defined spatial properties	The capability to support tumor cells at different differentiation stages while mimicking the tumor microenvironment	Tumor tissue with minimal manipulation
Specific disadvantages	Tends to merge into conglomerates, with challenges in size control	Many cells are needed for modeling	Modeling requires a substantial number of cells and advanced equipment	The efficiency of production is approximately 60–70%	Extended in vitro cultivation necessitates the use of supporting matrices or microfluidic devices
Mechanical properties	Variable, often lacking robust mechanical stability	Can be engineered to match specific mechanical properties (e.g., stiffness, elasticity)	Can be adjusted based on the bioink and printing conditions	Are often similar to native tissue	Preserved in the native tissue

## Data Availability

This study is based on previously published data, which are cited in the references. No new data were generated.

## Abbreviations

The following abbreviations are used in this manuscript:

AFM	Atomic Force Microscopy
AI	Artificial Intelligence
ATRA	All-Trans Retinoic Acid
CAFs	Cancer-Associated Fibroblasts
CAL	Cancer-Associated Line
CPCR	G Protein-Coupled Receptor
DL	Deep Learning
DMT Model	Derjaguin–Muller–Toporov Model
E	Young’s Modulus
ECM	Extracellular Matrix
EGFR	Epidermal Growth Factor Receptor
ENT	Ear, Nose, Throat
EPC2	Esophageal Progenitor Cell 2
GAG	Glycosaminoglycan
HA	Hyaluronic Acid
HEMa	Human Epidermal Melanocytes, adult
HLEC	Human Lymphatic Endothelial Cell
HNCs	Head and Neck Cancers
HNSCC	Head and Neck Squamous Cell Carcinoma
ICC	Intraclass Correlation Coefficient
JNK	c-Jun N-Terminal Kinase
MDA	Anderson Cancer Center
MDSCs	Myeloid-Derived Suppressor Cells
ML	Machine Learning
MOrgAna	ML-based Organoid Analysis Software
NECIT	Nano-Electro-Chemo-Immuno Therapy
OKF	Oral Keratinocyte Four
OSCC	Oral Squamous Cell Carcinoma
PCL	Poly-ε-caprolactone
PDE	Patient-Derived Tumor Explant
PEG	Polyethylene Glycol
PEGPH20	Pegylated Recombinant Human Hyaluronidase
PLCL	Poly(L-lactide-co-ε-caprolactone)
PLG	Poly(lactide-co-glycolide)
PVA	Polyvinyl Alcohol
SCC	Squamous Cell Carcinoma
TAMs	Tumor-Associated Macrophages
TME	Tumor Microenvironment
Tregs	T Cells
VGP	Vertical Growth Phase
